# Relationship between Egg Consumption and Body Composition as Well as Serum Cholesterol Level: Korea National Health and Nutrition Examination Survey 2008–2011

**DOI:** 10.3390/jcm10245918

**Published:** 2021-12-16

**Authors:** Jung-Eun Shim, Young-Gyun Seo

**Affiliations:** Department of Family Medicine, Hallym University Sacred Heart Hospital, Anyang-si 14068, Gyeonggi-do, Korea; nunnuv@naver.com

**Keywords:** eggs, lipids, fat mass, fat-free mass, muscle, food frequency questionnaire, 24-h dietary recall

## Abstract

We analyzed the relationship between egg consumption, body composition, and serum cholesterol levels. We obtained data on egg consumption by using a food frequency questionnaire (FFQ) (13,132 adults) and the 24-h dietary recall (24HR) (13,366 adults) from the fourth and fifth Korea National Health and Nutrition Examination Surveys (2008–2011). In men, consuming 2–3 eggs/week was associated with higher fat mass (FM), percentage body fat (PBF), and fat-to-muscle ratio (FtoM), compared to consuming <1 egg/week. In women, consuming 1–6 eggs/week was associated with higher low-density lipoprotein cholesterol, consuming 2–6 eggs/week was associated with higher total cholesterol, and consuming 4–6 eggs/week was associated with higher FM and high-density lipoprotein cholesterol, compared to consuming <1 egg/week. There was no relationship between egg consumption and the prevalence of dyslipidemia, and there was no relationship between egg consumption, body composition, and serum cholesterol levels according to the 24HR. However, there was some association with other cardiovascular diseases and consumption of certain amounts of eggs. Egg consumption investigated by FFQ was associated with body composition and serum cholesterol levels. However, the egg consumption investigated by the 24HR resulted in no health benefit or harm with respect to body composition and cholesterol.

## 1. Introduction

Eggs are one of the most consumed food groups worldwide [1,2]. They contain various proteins, lipids, vitamins, and minerals. In particular, eggs contain high-quality protein rich in various amino acids that promote protein synthesis. One large egg contains up to 6.3 g of protein, which provides antibacterial and immunoprotective properties to the human body [2]. Therefore, it is plausible that egg consumption may influence body composition. However, studies on egg consumption and body composition have seldom been reported. Liu et al. showed that excessive body fat according to egg intake did not change in men but decreased in women in Chinese adults [3]. Previous studies examining the risk of metabolic syndrome according to egg consumption also examined the changes in waist circumference (WC) and body mass index (BMI) according to egg consumption, but there were no effects of egg consumption on body compositions [4,5,6,7,8].

In addition to proteins, each egg contains 200–275 mg of cholesterol, making it one of the main sources of dietary cholesterol intake [9]. Studies on whether dietary cholesterol intake affects blood cholesterol and lipid levels have shown inconsistent results [10,11,12]. The effects of egg consumption on serum cholesterol and lipid levels have also been reported, but the results were also inconsistent, as follows: (1) there was no significant change in serum low-density lipoprotein cholesterol (LDL-C), high-density lipoprotein cholesterol (HDL-C), and triglyceride (TG) levels according to egg intake [4]; (2) the higher the frequency of egg consumption, the higher the serum total cholesterol (TC) level [5]; (3) serum LDL-C and HDL-C concentrations tended to increase in proportion to egg intake [13]. Guidelines regarding daily cholesterol intake also present conflicting recommendations. The 2000 American Heart Association guidelines recommend less than 300 mg/day of TC consumption, which is equivalent to 1 or 1 and a half eggs per day [14]. In contrast, restrictions on dietary egg consumption have been removed from the 2015–2020 Dietary Guidelines for Americans [15,16]. The American Heart Association, British Heart Foundation, Australian Heart Foundation, and New Zealand Heart Foundation have recently relaxed restrictions on egg consumption [17,18].

Therefore, in this study, we analyzed the differences in serum cholesterol concentration and body composition distribution according to egg consumption in adults. Although most previous studies investigated egg intake using the food frequency questionnaire (FFQ), this study investigated egg consumption using both FFQ and the 24-h dietary recall (24HR).

## 2. Materials and Methods

### 2.1. Study Population

This study obtained data from the 4th and 5th Korea National Health and Nutrition Examination Surveys (KNHANES), which is a nationally representative survey conducted between 2008 and 2011 by the Korea Disease Control and Prevention Agency. To assess the relationship between serum cholesterol level, body composition, and egg consumption, data from 18,915 participants aged ≥19 years who underwent dual-energy X-ray absorptiometry (DXA) were examined. Participants who had severe declines in kidney function (estimated glomerular filtration rate <30 mL/min/1.73 m^2^), a history of cancer diagnosis, inappropriate fasting duration before examination (>24 h or <8 h), inappropriate nutritional intake (<500 kcal/day or >5000 kcal/day), inappropriate water intake per body weight (≥90 g/kg), and missing data in questionnaire records or unavailable test results were excluded from the analyses. We obtained data on egg consumption by using FFQ from 13,132 participants (5407 men and 7725 women) and 24HR from 13,366 participants (5522 men and 7844 women).

All procedures were approved by the ethics committee of the Korea Disease Control and Prevention Agency (Approval Number: 2011-02CON-06-C, 2010-02CON-21-C, 2009-01CON-03-2C, and 2008-04EXP-01-C), and the study was carried out in accordance with the ethical standards laid down in the 1964 Declaration of Helsinki and its later amendments. Signed informed consent was obtained from all KNHANES participants. The KNHANES data were publicly available.

### 2.2. Assessment of Egg Consumption

Diet was assessed using a self-administered FFQ and the 24HR. The FFQ consists of a list of 63 commonly consumed food items in Korea. Each food intake has ten responses (almost never, 6–11 times/year, 1 time/month, 2–3 times/month, 1 time/week, 2–3 times/week, 4–6 times/week, 1 time/day, 2 times/day, and 3 times/day). We re-categorized the frequency of egg consumption as <1 time/week, 1 time/week, 2–3 times/week, 4–6 times/week, and ≥1 time/day [5]. The 24HR made participants self-report the type and amount of food consumed in the past 24 h. We categorized egg consumption as “not consumed” and “consumed” by using data from 24HR.

### 2.3. Assessment of Body Composition and Serum Cholesterol Level

Body composition was measured using DXA (Hologic Discovery, Hologic, Marlborough, MA, USA), including values for bone mineral content (BMC), fat mass (FM), fat-free mass (FFM), and percentage body fat (PBF) of the whole body and six regions (head, left arm, right arm, trunk, left leg, and right leg). Whole-body total FFM was calculated by subtracting the BMC from FFM. The fat-to-muscle ratio (FtoM) was calculated as the whole-body total FM divided by the whole-body total FFM. We defined obesity as BMI ≥25 kg/m^2^ [19], abdominal obesity as WC ≥90 cm in men and WC ≥85 in women [19], and obesity according to PBF as PBF ≥25% in men and PBF ≥30% in women [20].

TC, TG, HDL-C, and LDL-C levels were measured using enzymatic methods (Hitachi Automatic Analyzer 7600, Hitachi, Tokyo, Japan). The abnormal levels of cholesterol were defined as TC ≥200 mg/dL [21], LDL-C ≥130 mg/dL [21], TG ≥150 mg/dL [19], and HDL-C <40 in men and <50 in women [19].

### 2.4. Variables

The general characteristics of the participants by sex were analyzed using data on age, FM, PBF, FFM, FtoM, BMI, WC, TC, TG, HDL-C, LDL-C, systolic blood pressure, daily energy intake (total energy intake, protein intake, and water intake per body weight), smoking (never, past, or current), alcohol consumption (<once/month or ≥once/month), physical activity (PA), education (0–6 years, 7–12 years, or ≥13 years), individual income quartiles, previous diagnosis of dyslipidemia, hypertension, diabetes mellitus, stroke, myocardial infarction, and angina pectoris by doctors, treatment for hypertension, and survey year. PA was described as metabolic equivalent (MET), or classified as low, moderate, or high PA based on the data processing and analysis guidelines of the International Physical Activity Questionnaire (IPAQ) [22]. We also calculated predicted 10-year risk of a first hard atherosclerotic cardiovascular disease (ASCVD) event [23].

### 2.5. Statistical Analysis

Statistical analyses were conducted using STATA version 14.0 (StataCorp., College Station, TX, USA), and statistical significance was defined as *p* < 0.05. The sampling design for the KNHANES used two-stage stratified cluster sampling rather than simple random sampling. Thus, when analyzing the data, weights were applied by reflecting the contents of this complex sampling design. The Shapiro–Wilk test was used to evaluate normality of the data [24]. The analysis was conducted by transforming the data to a logarithmic scale to achieve a bell-shaped (approximately normal) distribution of the required variables. Linear regression analyses and chi-square tests were used for the comparative analysis of general characteristics by sex. Linear regression analyses were performed to analyze the relationship between body composition and serum cholesterol levels and egg consumption. Logistic regression analyses were performed to compare the prevalence of dyslipidemia, hypertension, diabetes mellitus, stroke, myocardial infarction, and angina pectoris according to egg consumption. Multivariable models were performed by adjusting for age, BMI, daily energy intake, smoking, alcohol consumption, PA, education, income, and survey year.

## 3. Results

### 3.1. General Characteristics of the Study Population by Sex

The mean age of the 13,366 participants whose data for 24HR were available was 44.43 years old, and 58.69% were women (Tables S1 and S2). Table 1 and Table 2 shows the general characteristics of the participants according to sex. Among the 13,132 participants whose data for FFQ were available, the mean age was 44.28 years old, and 58.86% were women. The mean FM (15.52 ± 0.12 vs. 19.01 ± 0.10), PBF (21.92 ± 0.13 vs. 32.85 ± 0.12), and FtoM (0.30 ± 0.002 vs. 0.53 ± 0.002) were higher in women than in men. FFM (51.21 ± 0.13 vs. 35.99 ± 0.08 1) was higher in men than in women. Mean TC (186.67 ± 0.61 vs. 185.80 ± 0.53), TG (124.58 ± 1.01 vs. 91.31 ± 1.01), and LDL-C (112.86 ± 0.96 vs. 109.96 ± 0.90) were higher and HDL-C (46.18 ± 0.20 vs. 51.30 ± 0.18) was lower in men than in women. The proportion of dyslipidemia diagnoses by doctors was higher in women than in men. The proportion of hypertension, diabetes mellitus, stroke, myocardial infarction, and angina pectoris diagnoses by doctors was higher in men than in women. In addition, ASCVD risk was higher in men than in women. WC, BMI, total energy intake, and water intake per body weight were higher in men than in women. The proportion of current smokers, alcohol consumers, participants with a high PA level, and highly educated participants was higher in men than in women.

The proportion of participants who consumed eggs more than once a day was higher in women than in men (7.42% vs. 8.17%) by using the FFQ; otherwise, in the 24HR, the proportion of participants who consumed eggs was slightly higher in men than in women (47.21% vs. 46.38%). In both methods, those who consumed more eggs tended to be younger and have more daily protein intake in both men and women. BMI, the proportion of obesity, and the proportion of obesity according to PBF in men were significantly different between groups and were higher in the group that consumed eggs compared to the group that did not consume eggs during the previous day. WC did not differ between the groups according to egg consumption in men. In women, PBF, BMI, WC, the proportion of obesity, and the proportion of abdominal obesity tended to decrease as egg consumption increased by both FFQ and 24HR.

TG in men and TC, TG, HDL-C, LDL-C, and the proportion of the abnormal levels of cholesterol in women were significantly different between groups by using the FFQ. The proportion of hypertension, diabetes mellitus, myocardial infarction, and angina pectoris diagnoses by doctors, and ASCVD risk in men and the proportion of dyslipidemia, hypertension, diabetes mellitus, stroke, myocardial infarction, and angina pectoris diagnoses by doctors, and ASCVD risk were significantly different between groups by using the FFQ. TG and the proportion of the abnormal levels of HDL-C in men and TC, TG, LDL-C, and the proportion of the abnormal levels of cholesterol in women were higher, and HDL-C in women was lower in the group that did not consume eggs compared to the group that consumed eggs during the previous day by using the FFQ. The proportion of hypertension, diabetes mellitus, stroke, myocardial infarction, and angina pectoris diagnoses by doctors, and ASCVD risk in men and the proportion of dyslipidemia, hypertension, diabetes mellitus, stroke, myocardial infarction, and angina pectoris diagnoses by doctors, and ASCVD risk in women were higher in the group that did not consume eggs compared to the group that consumed eggs during the previous day by using the FFQ.

Men who consumed more eggs tended to have more nutritional intake, to be current smokers, to be physically active, to be highly educated, and to earn higher income by FFQ. However, by using the 24HR, men who consumed one or more eggs per day tended to have more nutritional intake, to be current smokers, to be alcohol consumers, to be highly educated, and to earn higher income than men who did not consume eggs. Women who consumed more eggs tended to have more nutritional intake, to be highly educated, and to earn higher income by FFQ. Unlike in men, women who consumed less eggs tended to be physically active. In the 24HR, the proportion of participants with more nutritional intake, alcohol consumption, high education, and high-income levels was higher among women who consumed eggs compared to those who did not consume eggs during the previous day. In the case of PA, as in the FFQ, the proportion of high PA levels was higher among women who did not consume eggs compared to those who consumed eggs during the past 24 h.

### 3.2. Association between Serum Cholesterol Level, Prevalence of Dyslipidemia, and Egg Consumption

In men, the amount of egg intake and levels of TC, TG, HDL-C, and LDL-C were not relevant after adjusting for potential confounding variables when using both FFQ (Table 3) and 24HR (Table S3). Those who consumed eggs 4–6 times per week had higher prevalence of diabetes mellitus and those who consumed eggs 2–3 times per week had higher prevalence of stroke and myocardial infarction compared to those who consumed less than 1 egg per week by FFQ. The prevalence of dyslipidemia, hypertension, diabetes mellitus, stroke, myocardial infarction, and angina pectoris was not associated with egg consumption after adjusting for potential confounding variables by the 24HR.

In women, those who had an egg 2–6 times per week had higher levels of TC than those who ate less than 1 egg per week. Those who consumed eggs 4–6 times per week had higher levels of HDL-C compared to those who consumed less than 1 egg per week by FFQ, and those who consumed eggs 1–6 times per week had higher LDL-C levels compared to those who consumed less than 1 egg per week by FFQ (Table 4). When using the 24HR, egg consumption and TC, TG, HDL-C, and LDL-C levels were not associated after adjusting for potential confounding variables (Table S4). Those who consumed eggs 2–3 times per week had higher prevalence of hypertension, those who consumed eggs 4–6 times per week had higher prevalence of diabetes mellitus, and those who consumed egg once per week had higher prevalence of angina pectoris compared to those who consumed less than 1 egg per week by FFQ. The prevalence of hypertension was higher among women who consumed eggs compared to those who did not consume eggs during the past 24 h.

### 3.3. Association between Body Composition, Waist Circumfernce, and Egg Consumption

In men, the FM, PBF, and FtoM were significantly greater in the group consuming 2–3 eggs per week compared to the group consuming less than one egg per week when using FFQ. There was no significant difference in FFM and WC according to egg consumption (Table 5). Furthermore, there was no significant correlation between FM, PBF, FFM, FtoM, and WC according to egg consumption in men by the 24HR (Table S5).

In women, the group consuming 4–6 eggs per week had higher FM than the group consuming less than one egg per week. There was no significant difference in PBF, FFM, FtoM, and WC according to egg consumption according to the FFQ (Table 6). By using the 24HR, there was no significant difference in FM, PBF, FFM, FtoM, and WC according to egg consumption (Table S6).

## 4. Discussion

The purpose of this study was to examine the relationship between egg consumption, serum cholesterol levels, and body composition distribution in Korean adult men and women using the 2008–2011 KNHANES. In men, consuming 2–3 eggs per week was associated with higher FM, PBF, FtoM, and prevalence of stroke and myocardial infarction, and consuming 4–6 eggs per week was associated with higher prevalence of diabetes mellitus than consuming less than one egg per week. In women, consuming 2–6 eggs per week was associated with higher TC, consuming 4–6 eggs per week was associated with higher HDL-C, FM, and prevalence of diabetes mellitus, consuming 1–6 eggs per week was associated with higher LDL-C, consuming 2–3 eggs per week was associated with higher prevalence of hypertension, and consuming 1 egg per week was associated with higher prevalence of angina pectoris, compared to consuming less than one egg per week. According to 24HR, there was no relationship between egg intake and health indicators, except for hypertension.

In two previous studies using the 2007–2008 KNHANES and the 2013 KNHANES data, similar trends were observed: higher egg intake was associated with younger age, higher education, and higher income levels [4,5]. This is consistent with the results of our study, and it can be inferred that the participants who are young, highly educated, and have higher income levels have an interest in a healthy diet, and it can be inferred that egg consumption naturally increased when the egg was recognized as a healthy food. According to Kim et al., subjects who frequently ate eggs tended to have higher intakes of protein and fat, as well as other nutrients such as calcium, phosphorus, and riboflavin. In addition, it was found that the higher the egg intake, the greater the PA [5].

In a meta-analysis [25], it was found that the LDL-C levels and LDL-C/HDL-C ratio increased in proportion to egg consumption. Another meta-analysis found that egg consumption increased TC, LDL-C, and HDL-C levels, but had no effect on LDL-C/HDL-C ratio [26]. However, a recent study reported that consuming more than 3 eggs per week was associated with lower LDL-C levels and LDL-C/HDL-C ratio compared to consuming up to one egg per week [27]. The effect of dietary cholesterol intake on blood cholesterol levels is limited [10,11,12]. In addition, the degree of response to dietary cholesterol may vary depending on various conditions, individual characteristics, and the degree of compensatory mechanisms such as suppression of cholesterol synthesis when a large amount of dietary cholesterol is consumed [5,11,28,29]. In this study, it was confirmed that egg intake in men and women had a relationship with TC, HDL-C, and LDL-C levels. Although there was some association between egg consumption and serum cholesterol levels in our study, a dose–response relationship was not established. This is no different from previous studies in which the effects and responses of cholesterol intake on blood cholesterol levels varied from person to person [5,11,28,29]. In this study, no relationship was found between the prevalence of dyslipidemia and egg consumption. However, there was some association with other cardiovascular diseases and consumption of certain amounts of eggs. In previous studies, it was reported that egg consumption has positive effects on metabolic syndrome [6,7,8], and a follow-up study on the relationship between egg consumption and the risk of dyslipidemia and metabolic syndrome is needed.

According to Liu et al., central obesity and excessive body fat were improved in proportion to egg consumption in women, but there was no significant change in men [3]. In our study, BMI and WC decreased according to egg consumption in women, but there was no significant change in men. However, in our study, FM, PBF, and FtoM in men and FM in women showed a tendency to increase in the egg intake group of a certain amount, whereas in the 24HR, there was no change in body composition distribution according to egg consumption in either men or women. Although the mechanism by which egg intake affects body composition is not clear, eggs are a food rich in protein and essential amino acids and are involved in protein synthesis [2], which is considered to be able to improve muscle mass, and it could be inferred that PBF would be reduced or not affected by egg consumption. However, in our study, FM, PBF, and FtoM showed a tendency to increase with egg consumption. It is considered that the nutrient intake in the specific amount of egg intake group was higher than that of the reference group. In addition, excessive PBF was calculated using the equation and then categorized in the study by Liu et al. [3], whereas the FM values of participants were measured by DXA in our study, which might be a factor contributing to the difference in the results. Studies on the effect of egg consumption on the distribution of body composition are still lacking. More detailed follow-up studies, including data on intake of other nutrients such as protein and fat, and data on the concentrations of various hormones involved in muscle and fat accumulation in the body are needed.

The strengths of this study are as follows: first, data from the KNHANES, which was extensively surveyed over four years on egg consumption in Korean adult men and women, were used. Since egg consumption differs from country to country in terms of recipes and dietary patterns, it is necessary to perform an analysis in each country. Second, body composition data directly measured by DXA, which is the gold standard, were used. Finally, both FFQ and 24HR were used. Most previous studies used FFQ to collect data on egg consumption [6,7,25]. FFQ data has the advantage of being able to investigate the amount or frequency of egg intake in more detail. However, it has the disadvantage of relying on inaccurate long-term memories of individuals when responding to questions. On the other hand, although information on the amount or frequency of egg intake collected through the 24HR is somewhat lacking compared to the FFQ, recall bias may be smaller as individuals recall and record food consumed within the past 24 h. Nevertheless, the 24HR may not fully reflect an individual’s usual eating habits, and thus may lead to biased results. In this regard, focusing on interpreting the results of FFQ, there is still no established dose–response relationship between egg intake and body composition distribution as well as serum cholesterol level, but it is thought to be related to the specific amount of egg intake.

This study had several limitations. First, the data based on questionnaires may have recall bias. Second, since it was a cross-sectional study, it had a limited ability to demonstrate a causal relationship. Finally, although the analysis was performed by adjusting for clinically meaningful variables, potential confounding factors that were not considered could not be excluded.

The current results can be generalized to all Koreans due to the large sample size, high response rate (about 80%), and the use of proportional systematic samples through multistage stratification according to region, sex, and age group. Although various lifestyle factors were included in this study, multicollinearity was not detected in the regression analysis in which smoking, alcohol consumption, and PA were included as covariates. Previous studies have also reported that smoking, alcohol consumption, and PA can independently influence body composition [30,31] and cholesterol levels [32]. In 2018, egg consumption worldwide was 9.68 kg/person/year, while egg consumption in Korea was 12.93 kg/person/year [33]. In other words, egg consumption was slightly higher than the global average. Therefore, the poor health results of this study can be emphasized in countries with similar or higher egg consumption than that of Korea, and the good health results of this study can be emphasized in countries with lower egg consumption than that of Korea.

## 5. Conclusions

In conclusion, this study found that egg consumption investigated by FFQ was related to body composition as well as serum cholesterol levels in Korean adult men and women. In men, body composition distributions were related to egg intake rather than serum cholesterol levels, and in women, serum cholesterol levels were related to egg intake rather than body composition distributions. Therefore, it is necessary to pay more attention to the advice on egg intake in men at high risk of obesity and women at high risk of elevated cholesterol. However, the egg consumption investigated by the 24HR resulted in no health benefit or harm in terms of cholesterol and body composition.

## Figures and Tables

**Table 1:** General characteristics according to egg consumption in men (food frequency questionnaire).

Characteristics		Total	<1/Week	1/Week	2–3/Week	4–6/Week	≥1/Day	*p*-Value ^a^
Age, years		44.15 ± 0.33	50.99 ± 0.60	44.98 ± 0.59	42.34 ± 0.40	39.59 ± 0.66	39.15 ± 0.86	<0.001
FM, kg		15.52 ± 0.12	14.85 ± 0.22	15.45 ± 0.22	15.56 ± 0.18	16.19 ± 0.25	15.4 ± 0.39	0.001
PBF, %		21.92 ± 0.13	21.60 ± 0.21	21.92 ± 0.23	21.89 ± 0.18	22.24 ± 0.24	21.95 ± 0.38	0.157
PBF ≥ 25%		249 (4.61)	40 (3.03)	59 (5.06)	83 (4.32)	36 (6.03)	31 (7.73)	<0.001
FFM, kg		51.21 ± 0.13	50.01 ± 0.26	51.02 ± 0.26	51.36 ± 0.19	52.68 ± 0.33	51.95 ± 0.38	<0.001
FtoM		0.30 ± 0.002	0.30 ± 0.003	0.30 ± 0.004	0.30 ± 0.003	0.31 ± 0.004	0.30 ± 0.006	0.125
BMI, kg/m^2^		24.03 ± 0.058	23.83 ± 0.13	23.99 ± 0.12	23.99 ± 0.090	24.41 ± 0.14	24.09 ± 0.21	0.034
BMI ≥ 25 kg/m^2^		1910 (35.32)	427 (32.30)	406 (34.85)	691 (35.95)	231 (38.69)	155 (38.65)	0.031
WC, cm		83.98 ± 0.17	84.29 ± 0.35	83.95 ± 0.36	83.63 ± 0.27	84.57 ± 0.41	83.67 ± 0.55	0.594
WC ≥ 90 cm		1424 (26.34)	359 (27.16)	313 (26.87)	486 (25.29)	165 (27.64)	101 (25.91)	0.642
TC, mg/dL		186.67 ± 0.61	185.57 ± 1.15	187.34 ± 1.21	186.98 ± 0.97	187.75 ± 1.69	183.10 ± 2.60	0.800
TC ≥ 200 mg/dL		1806 (33.93)	421 (32.74)	380 (33.13)	658 (34.60)	211 (35.70)	136 (34.34)	0.667
TG, mg/dL ^b^		124.58 ± 1.01	129.47 ± 1.02	123.94 ± 1.02	125.47 ± 1.02	119.27 ± 1.03	115.06 ± 1.04	0.007
TG ≥ 150 mg/dL		1957 (36.72)	493 (38.25)	426 (37.04)	688 (36.15)	207 (34.97)	143 (36.11)	0.653
HDL-C, mg/dL		46.18 ± 0.20	46.15 ± 0.42	45.81 ± 0.38	46.15 ± 0.31	46.72 ± 0.57	46.72 ± 0.55	0.224
HDL-C < 40 mg/dL		1786 (33.56)	466 (36.24)	388 (33.83)	613 (32.23)	197 (33.33)	122 (30.81)	0.134
LDL-C, mg/dL		112.86 ± 0.96	112.32 ± 2.11	113.22 ± 2.24	112.64 ± 1.54	117.27 ± 2.73	103.98 ± 3.55	0.610
LDL-C ≥ 130 mg/dL		429 (27.59)	95 (27.94)	85 (26.40)	168 (28.52)	61 (31.28)	20 (18.35)	0.165
Dyslipidemia		446 (8.25)	131 (9.91)	94 (8.07)	143 (7.44)	46 (7.71)	32 (7.98)	0.147
Hypertension		1210 (22.38)	399 (30.18)	252 (21.63)	382 (19.88)	98 (16.42)	79 (19.70)	<0.001
Diabetes mellitus		463 (8.56)	167 (12.63)	102 (8.76)	136 (7.08)	29 (4.86)	29 (7.23)	<0.001
Stroke		128 (2.37)	41 (3.10)	26 (2.23)	42 (2.19)	8 (1.34)	11 (2.74)	0.170
Myocardial infarction		55 (1.02)	20 (1.51)	15 (1.29)	10 (0.52)	4 (0.67)	6 (1.50)	0.034
Angina pectoris		99 (1.83)	41 (3.10)	19 (1.63)	24 (1.25)	10 (1.68)	5 (1.25)	0.002
Predicted 10-year risk of a first hard ASCVD event		0.065 ± 0.001	0.10 ± 0.004	0.067 ± 0.002	0.054 ± 0.002	0.042 ± 0.002	0.046 ± 0.004	<0.001
Nutritional intake								
	Total energy intake, kcal/day	2327.45 ± 14.96	2142.46 ± 28.74	2301.03 ± 29.13	2385.47 ± 22.82	2462.37 ± 41.97	2439.92 ± 57.54	<0.001
	Protein intake, g/day	84.71 ± 0.77	74.39 ± 1.30	84.12 ± 1.74	87.42 ± 1.04	90.96 ± 2.05	91.86 ± 2.75	<0.001
	Water intake/body weight, g/kg/day	16.38 ± 0.17	14.83 ± 0.32	16.21 ± 0.36	16.81 ± 0.26	17.68 ± 0.49	17.04 ± 0.60	<0.001
Smoking								<0.001
	None	1221 (22.58)	299 (22.62)	263 (22.58)	414 (21.54)	155 (25.96)	90 (22.44)	
	Past	1987 (36.75)	525 (39.71)	465 (39.91)	676 (35.17)	187 (31.32)	134 (33.42)	
	Current	2199 (40.67)	498 (37.67)	437 (37.51)	832 (43.29)	255 (42.71)	177 (44.14)	
Alcohol drinking								<0.001
	<1 time/month	1384 (25.60)	416 (31.47)	295 (25.32)	409 (21.28)	155 (25.96)	109 (27.18)	
	≥1 time/month	4023 (74.40)	906 (68.53)	870 (74.68)	1513 (78.72)	442 (74.04)	292 (72.82)	
Physical activity								0.035
	Low	1503 (27.80)	400 (30.26)	320 (27.47)	528 (27.47)	167 (27.97)	88 (21.95)	
	Moderate	2100 (38.84)	472 (35.70)	472 (40.52)	767 (39.91)	228 (38.19)	161 (40.15)	
	High	1804 (33.36)	450 (34.04)	373 (32.02)	627 (32.62)	202 (33.84)	152 (37.91)	
Education								<0.001
	0–6 years	864 (15.98)	416 (31.47)	184 (15.79)	185 (9.63)	48 (8.04)	31 (7.73)	
	7–12 years	2266 (41.91)	571 (43.19)	505 (43.35)	839 (43.65)	214 (35.85)	137 (34.16)	
	≥13 years	2277 (42.11)	335 (25.34)	476 (40.86)	898 (46.72)	335 (36.11)	233 (58.10)	
Income								<0.001
	Q1	1277 (23.62)	377 (28.52)	263 (22.58)	417 (21.70)	135 (22.61)	85 (21.20)	
	Q2	1398 (25.86)	361 (27.31)	292 (25.06)	499 (25.96)	160 (26.80)	86 (21.45)	
	Q3	1365 (25.25)	317 (23.98)	299 (25.67)	488 (25.39)	145 (24.29)	116 (28.93)	
	Q4	1367 (25.28)	267 (20.20)	311 (26.70)	518 (26.95)	157 (26.30)	114 (28.43)	
Survey year								<0.001
	2008	941 (17.40)	252 (19.06)	229 (19.66)	310 (16.13)	82 (13.74)	68 (16.96)	
	2009	2134 (39.47)	518 (39.18)	473 (40.60)	745 (38.76)	223 (37.35)	175 (43.64)	
	2010	1657 (30.65)	393 (29.73)	348 (29.87)	614 (31.95)	187 (31.32)	115 (28.68)	
	2011	675 (12.48)	159 (12.03)	115 (9.87)	253 (13.16)	105 (17.59)	43 (10.72)	

FM, fat mass; PBF; percentage body fat; FFM, fat-free mass; FtoM, fat-to-muscle ratio; BMI, body mass index; WC, waist circumference; TC, total cholesterol; TG, triglyceride; HDL-C, high-density lipoprotein cholesterol; LDL-C, low-density lipoprotein cholesterol; ASCVD, atherosclerotic cardiovascular disease. Data are presented as means ± standard error for continuous variables and numbers (%) for categorical variables. ^a^ *p*-value from linear regression analysis for continuous variables or χ^2^ test for categorical variables, comparing differences between two groups. ^b^ Geometric mean ± standard error.

**Table 2:** General characteristics according to egg consumption in women (food frequency questionnaire).

Characteristics		Total	<1/Week	1/Week	2–3/Week	4–6/Week	≥1/Day	*p*-Value ^a^
Age, years		44.69 ± 0.28	53.60 ± 0.53	44.54 ± 0.49	41.99 ± 0.36	39.33 ± 0.47	39.05 ± 0.60	<0.001
FM, kg		19.01 ± 0.10	19.31 ± 0.18	18.98 ± 0.17	18.91 ± 0.15	19.15 ± 0.25	18.49 ± 0.28	0.043
PBF, %		32.85 ± 0.12	33.38 ± 0.20	32.94 ± 0.18	32.61 ± 0.16	32.65 ± 0.25	32.34 ± 0.27	<0.001
PBF ≥ 30%		257 (3.33)	67 (3.27)	52 (3.58)	81 (3.18)	40 (3.81)	17 (2.69)	0.732
FFM, kg		35.99 ± 0.081	35.81 ± 0.14	35.89 ± 0.14	36.16 ± 0.12	36.38 ± 0.18	35.74 ± 0.22	0.091
FtoM		0.53 ± 0.002	0.54 ± 0.005	0.53 ± 0.004	0.52 ± 0.003	0.53 ± 0.006	0.52 ± 0.006	0.001
BMI, kg/m^2^		23.20 ± 0.058	23.88 ± 0.11	23.16 ± 0.11	23.01 ± 0.09	22.97 ± 0.15	22.57 ± 0.17	<0.001
BMI ≥ 25 kg/m^2^		2196 (28.43)	719 (35.14)	416 (28.61)	672 (26.40)	255 (24.31)	134 (21.24)	<0.001
WC, cm		77.68 ± 0.18	80.31 ± 0.31	77.60 ± 0.32	77.03 ± 0.27	76.51 ± 0.39	75.18 ± 0.47	<0.001
WC ≥ 85 cm		1947 (25.20)	698 (34.12)	355 (24.42)	595 (23.38)	204 (19.45)	95 (15.06)	<0.001
TC, mg/dL		185.80 ± 0.53	190.72 ± 0.92	185.12 ± 1.08	184.51 ± 0.85	184.08 ± 1.21	181.06 ± 1.77	<0.001
TC ≥ 200 mg/dL		2626 (34.90)	830 (42.22)	487 (34.22)	794 (31.90)	324 (31.40)	191 (31.06)	<0.001
TG, mg/dL ^b^		91.31 ± 1.01	103.23 ± 1.02	90.66 ± 1.02	88.74 ± 1.01	85.11 ± 1.02	82.67 ± 1.03	<0.001
TG ≥ 150 mg/dL		1550 (20.56)	568 (28.79)	294 (20.63)	453 (18.18)	153 (14.81)	82 (13.33)	<0.001
HDL-C, mg/dL		51.30 ± 0.18	49.29 ± 0.32	50.72 ± 0.37	51.91 ± 0.28	53.23 ± 0.45	52.36 ± 0.54	<0.001
HDL-C < 50 mg/dL		3885 (51.63)	1181 (60.07)	741 (52.07)	1214 (48.77)	462 (44.77)	287 (46.67)	<0.001
LDL-C, mg/dL		109.96 ± 0.90	111.46 ± 1.86	110.52 ± 1.95	109.87 ± 1.40	110.90 ± 2.50	99.91 ± 3.34	0.029
LDL-C ≥ 130 mg/dL		453 (26.22)	122 (30.35)	90 (26.87)	135 (22.61)	76 (29.34)	30 (22.22)	0.040
Dyslipidemia		749 (9.70)	288 (14.08)	158 (10.87)	204 (8.02)	62 (5.91)	37 (5.86)	<0.001
Hypertension		1472 (19.06)	695 (33.97)	277 (19.05)	358 (14.07)	98 (9.34)	44 (6.97)	<0.001
Diabetes mellitus		494 (6.39)	238 (11.63)	83 (5.71)	132 (5.19)	20 (1.91)	21 (3.33)	<0.001
Stroke		112 (1.45)	62 (3.03)	21 (1.44)	22 (0.86)	5 (0.48)	2 (0.32)	<0.001
Myocardial infarction		35 (0.45)	16 (0.78)	8 (0.55)	9 (0.35)	2 (0.19)	0 (0.00)	0.037
Angina pectoris		101 (1.31)	42 (2.05)	25 (1.72)	18 (0.71)	9 (0.86)	7 (1.11)	0.001
Predicted 10-year risk of a first hard ASCVD event		0.035 ± 0.001	0.070 ± 0.002	0.032 ± 0.002	0.023 ± 0.001	0.017 ± 0.001	0.015 ± 0.001	<0.001
Nutritional intake								
	Total energy intake, kcal/day	1670.27 ± 9.70	1565.52 ± 17.30	1641.73 ± 19.09	1710.32 ± 16.81	1758.32 ± 23.77	1741.52 ± 30.06	<0.001
	Protein intake, g/day	59.55 ± 0.46	52.06 ± 0.75	59.12 ± 0.87	61.63 ± 0.76	64.11 ± 1.15	66.23 ± 1.56	<0.001
	Water intake/body weight, g/kg/day	15.06 ± 0.16	13.14 ± 0.27	14.71 ± 0.31	15.80 ± 0.24	16.41 ± 0.36	16.22 ± 0.42	<0.001
Smoking								0.754
	None	7066 (91.47)	1873 (91.54)	1337 (91.95)	2330 (91.55)	950 (90.56)	576 (91.28)	
	Past	290 (3.75)	82 (4.01)	54 (3.71)	88 (3.46)	46 (4.39)	20 (3.17)	
	Current	369 (4.78)	91 (4.45)	63 (4.33)	127 (4.99)	53 (5.05)	35 (5.55)	
Alcohol drinking								<0.001
	<1 time/month	4656 (60.27)	1429 (69.84)	882 (60.66)	1400 (55.01)	591 (56.34)	354 (56.10)	
	≥1 time/month	3069 (39.73)	617 (30.16)	572 (39.34)	1145 (44.99)	458 (43.66)	277 (43.90)	
Physical activity								0.034
	Low	2543 (32.92)	650 (31.77)	488 (33.56)	832 (32.69)	370 (35.27)	203 (32.17)	
	Moderate	3310 (42.85)	853 (41.69)	600 (41.27)	1118 (43.93)	446 (42.52)	293 (46.43)	
	High	1872 (24.23)	543 (26.54)	366 (25.17)	595 (23.38)	233 (22.21)	135 (21.39)	
Education								<0.001
	0–6 years	2204 (28.53)	1149 (56.16)	402 (27.65)	498 (19.57)	97 (9.25)	58 (9.19)	
	7–12 years	3103 (40.17)	624 (30.50)	626 (43.05)	1111 (43.65)	457 (43.57)	285 (45.17)	
	≥13 years	2418 (31.30)	273 (13.34)	426 (29.30)	936 (36.78)	495 (47.19)	288 (45.64)	
Income								<0.001
	Q1	1869 (24.19)	591 (28.89)	352 (24.21)	569 (22.36)	231 (22.02)	126 (19.97)	
	Q2	1978 (25.61)	565 (27.61)	368 (25.31)	624 (24.52)	265 (25.26)	156 (24.72)	
	Q3	1985 (25.70)	472 (23.07)	375 (25.79)	705 (27.70)	269 (25.64)	164 (25.99)	
	Q4	1893 (24.50)	418 (20.43)	359 (24.69)	647 (25.42)	284 (27.07)	185 (29.32)	
Survey year								0.055
	2008	1477 (19.12)	406 (19.84)	286 (19.67)	470 (18.47)	195 (18.59)	120 (19.02)	
	2009	2936 (38.01)	804 (39.30)	559 (38.45)	948 (37.25)	371 (35.37)	254 (40.25)	
	2010	2345 (30.36)	597 (29.18)	425 (29.23)	821 (32.26)	319 (30.41)	183 (29.00)	
	2011	967 (12.52)	239 (11.68)	184 (12.65)	306 (12.02)	164 (15.63)	74 (11.73)	

FM, fat mass; PBF; percentage body fat; FFM, fat-free mass; FtoM, fat-to-muscle ratio; BMI, body mass index; WC, waist circumference; TC, total cholesterol; TG, triglyceride; HDL-C, high-density lipoprotein cholesterol; LDL-C, low-density lipoprotein cholesterol; ASCVD, atherosclerotic cardiovascular disease. Data are presented as means ± standard error for continuous variables and numbers (%) for categorical variables. ^a^ *p*-value from linear regression analysis for continuous variables or χ^2^ test for categorical variables, comparing differences between two groups. ^b^ Geometric mean ± standard error.

**Table 3:** Association between serum cholesterol level, prevalence of dyslipidemia, and egg consumption in men (food frequency questionnaire).

Sex	Outcome	Characteristics		Crude	Age-Adjusted	Multivariable ^a^	*p*-Value ^a^
Men	TC	Egg consumption					
			<1/week	reference	reference	reference	reference
			1/week	1.76 ± 1.61	3.45 ± 1.59	2.43 ± 1.56	0.120
			2–3/week	1.41 ± 1.49	3.82 ± 1.48	2.56 ± 1.48	0.084
			4–6/week	2.18 ± 2.08	5.42 ± 2.10	3.37 ± 2.06	0.100
			≥1/day	−2.47 ± 2.82	0.85 ± 2.76	−0.35 ± 2.63	0.895
	TG ^b^	Egg consumption					
			<1/week	reference	reference	reference	reference
			1/week	−0.044 ± 0.031	−0.007 ± 0.030	−0.025 ± 0.028	0.374
			2–3/week	−0.031 ± 0.027	0.020 ± 0.026	−0.006 ± 0.026	0.798
			4–6/week	0.08 ± 0.034	−0.013 ± 0.034	−0.055 ± 0.033	0.099
			≥1/day	−0.12 ± 0.049	−0.047 ± 0.047	−0.059 ± 0.043	0.168
	HDL-C	Egg consumption					
			<1/week	reference	reference	reference	reference
			1/week	−0.34 ± 0.55	−0.71 ± 0.55	−0.47 ± 0.52	0.359
			2–3/week	−0.0005 ± 0.49	−0.54 ± 0.49	−0.59 ± 0.48	0.222
			4–6/week	0.57 ± 0.67	−0.15 ± 0.68	0.21 ± 0.67	0.756
			≥1/day	0.57 ± 0.69	−0.17 ± 0.68	0.096 ± 0.65	0.882
	LDL-C	Egg consumption					
			<1/week	reference	reference	reference	reference
			1/week	0.90 ± 3.06	2.41 + 3.14	1.02 ± 3.00	0.734
			2–3/week	0.32 ± 2.64	2.33 + 2.71	1.09 ± 2.67	0.682
			4–6/week	4.95 ± 3.48	7.52 + 3.56	4.37 ± 3.54	0.218
			≥1/day	−8.34 ± 4.07	−5.35 + 3.95	−5.44 ± 3.82	0.155
	Dyslipidemia	Egg consumption					
			<1/week	reference	reference	reference	reference
			1/week	0.70 (0.49 to 1.00)	0.89 (0.61 to 1.29)	0.77 (0.53 to 1.12)	0.176
			2–3/week	0.69 (0.50 to 0.95)	0.99 (0.71 to 1.38)	0.85 (0.61 to 1.20)	0.362
			4–6/week	0.63 (0.42 to 0.96)	1.01 (0.65 to 1.56)	0.84 (0.54 to 1.30)	0.434
			≥1/day	0.60 (0.36 to 0.98)	0.95 (0.56 to 1.59)	0.76 (0.44 to 1.31)	0.324
	Hypertension	Egg consumption					
			<1/week	reference	reference	reference	reference
			1/week	0.67 (0.54 to 0.83)	1.01 (0.79 to 1.30)	0.91 (0.71 to 1.18)	0.487
			2–3/week	0.58 (0.48 to 0.71)	1.12 (0.89 to 1.40)	1.04 (0.82 to 1.32)	0.737
			4–6/week	0.47 (0.35 to 0.62)	1.05 (0.76 to 1.46)	0.97 (0.70 to 1.35)	0.872
			≥1/day	0.55 (0.40 to 0.77)	1.28 (0.90 to 1.84)	1.13 (0.77 to 1.66)	0.542
	Diabetes mellitus	Egg consumption					
			<1/week	reference	reference	reference	reference
			1/week	0.57 (0.42 to 0.76)	0.81 (0.59 to 1.12)	0.78 (0.57 to 1.09)	0.147
			2–3/week	0.47 (0.36 to 0.62)	0.82 (0.60 to 1.11)	0.83 (0.61 to 1.13)	0.242
			4–6/week	0.21 (0.13 to 0.35)	0.42 (0.26 to 0.70)	0.42 (0.25 to 0.70)	0.001
			≥1/day	0.49 (0.25 to 0.65)	0.80 (0.48 to 1.33)	0.80 (0.48 to 1.35)	0.405
	Stroke	Egg consumption					
			<1/week	reference	reference	reference	reference
			1/week	0.68 (0.39 to 1.20)	1.11 (0.61 to 2.01)	1.19 (0.63 to 2.25)	0.598
			2–3/week	0.75 (0.46 to 1.24)	1.61 (0.95 to 2.72)	1.86 (1.08 to 3.22)	0.026
			4–6/week	0.44 (0.17 to 1.14)	1.13 (0.43 to 2.99)	1.35 (0.51 to 3.62)	0.545
			≥1/day	0.72 (0.26 to 2.01)	1.76 (0.61 to 5.07)	2.15 (0.72 to 6.43)	0.171
	Myocardial infarction	Egg consumption					
			<1/week	reference	reference	reference	reference
			1/week	0.74 (0.30 to 1.84)	1.14 (0.44 to 2.91)	1.05 (0.39 to 2.85)	0.924
			2–3/week	0.20 (0.09 to 0.47)	0.39 (0.16 to 0.96)	0.41 (0.17 to 0.98)	0.045
			4–6/week	0.68 (0.17 to 2.77)	1.57 (0.43 to 5.74)	1.68 (0.45 to 6.19)	0.439
			≥1/day	0.85 (0.26 to 2.71)	1.88 (0.56 to 6.30)	1.98 (0.53 to 7.34)	0.307
	Angina pectoris	Egg consumption					
			<1/week	reference	reference	reference	reference
			1/week	0.58 (0.30 to 1.14)	0.92 (0.47 to 1.81)	0.82 (0.41 to 1.61)	0.559
			2–3/week	0.37 (0.21 to 0.66)	0.74 (0.41 to 1.35)	0.64 (0.36 to 1.16)	0.140
			4–6/week	0.47 (0.21 to 1.04)	1.14 (0.50 to 2.61)	0.95 (0.40 to 2.25)	0.913
			≥1/day	0.02 (0.015 to 0.029)	0.75 (0.25 to 2.27)	0.6 (0.20 to 1.84)	0.375

TC, total cholesterol; TG, triglyceride; HDL-C, high-density lipoprotein cholesterol; LDL-C, low-density lipoprotein cholesterol. Data are presented as beta coefficient ± standard error or odds ratio (95% confidence interval). ^a^ Multivariable linear (TC, TG, HDL-C, and LDL-C) or logistic (dyslipidemia, hypertension, diabetes mellitus, stroke, myocardial infarction, and angina pectoris) regression model adjusted for age, body mass index status, total energy intake, protein intake, water intake per body weight, smoking, alcohol drinking, physical activity, education, income, and survey year. ^b^ Log-transformed.

**Table 4:** Association between serum cholesterol level, prevalence of dyslipidemia, and egg consumption in women (food frequency questionnaire).

Sex	Outcome	Characteristics		Crude	Age-Adjusted	Multivariable ^a^	*p*-Value ^a^
Women	TC	Egg consumption					
			<1/week	reference	reference	reference	reference
			1/week	−5.60 ± 1.44	1.61 ± 1.39	1.66 ± 1.38	0.231
			2–3/week	−6.21 ± 1.19	3.10 ± 1.22	3.21 ± 1.23	0.009
			4–6/week	−6.65 ± 1.50	4.72 ± 1.46	4.51 ± 1.44	0.002
			≥1/day	−9.66 ± 1.94	2.18 ± 1.93	2.69 ± 1.91	0.160
	TG ^b^	Egg consumption					
			<1/week	reference	reference	reference	reference
			1/week	−0.13 ± 0.023	−0.012 ± 0.022	0.005 ± 0.021	0.810
			2–3/week	−0.15 ± 0.020	0.0006 ± 0.020	0.026 ± 0.019	0.176
			4–6/week	−0.19 ± 0.27	−0.007 ± 0.026	0.014 ± 0.026	0.598
			≥1/day	−0.22 ± 0.030	−0.029 ± 0.030	0.011 ± 0.029	0.707
	HDL-C	Egg consumption					
			<1/week	reference	reference	reference	reference
			1/week	1.43 ± 0.48	0.19 ± 0.48	−0.093 ± 0.46	0.840
			2–3/week	2.62 ± 0.41	1.02 ± 0.41	0.59 ± 0.40	0.143
			4–6/week	3.94 ± 0.56	1.98 ± 0.55	1.61 ± 0.53	0.003
			≥1/day	3.07 ± 0.63	1.03 ± 0.65	0.59 ± 0.62	0.345
	LDL-C	Egg consumption					
			<1/week	reference	reference	reference	reference
			1/week	−0.94 ± 2.94	4.48 ± 2.79	5.24 ± 2.59	0.044
			2–3/week	−1.58 ± 2.24	3.57 ± 2.13	4.17 ± 2.00	0.038
			4–6/week	−0.56 ± 3.06	5.63 ± 2.83	5.73 ± 2.79	0.041
			≥1/day	−11.55 ± 3.83	−4.07 ± 3.72	−1.32 ± 3.58	0.713
	Dyslipidemia	Egg consumption					
			<1/week	reference	reference	reference	reference
			1/week	0.74 (0.57 to 0.96)	1.28 (0.96 to 1.71)	1.21 (0.91 to 1.62)	0.195
			2–3/week	0.5 (0.39 to 0.63)	1.02 (0.79 to 1.32)	0.96 (0.74 to 1.25)	0.784
			4–6/week	0.41 (0.29 to 0.59)	1.03 (0.70 to 1.52)	0.99 (0.66 to 1.48)	0.970
			≥1/day	0.41 (0.27 to 0.63)	1.07 (0.69 to 1.67)	1.03 (0.64 to 1.64)	0.913
	Hypertension	Egg consumption					
			<1/week	reference	reference	reference	reference
			1/week	0.47 (0.39 to 0.56)	1.01 (0.80 to 1.28)	1.01 (0.80 to 1.29)	0.908
			2–3/week	0.29 (0.24 to 0.34)	0.78 (0.63 to 0.97)	0.79 (0.64 to 0.98)	0.035
			4–6/week	0.19 (0.14 to 0.25)	0.69 (0.49 to 0.96)	0.77 (0.54 to 1.10)	0.151
			≥1/day	0.15 (0.10 to 0.23)	0.56 (0.37 to 0.84)	0.6 (0.39 to 0.90)	0.015
	Diabetes mellitus	Egg consumption					
			<1/week	reference	reference	reference	reference
			1/week	0.45 (0.33 to 0.61)	0.79 (0.57 to 1.10)	0.83 (0.60 to 1.15)	0.252
			2–3/week	0.42 (0.32 to 0.55)	0.92 (0.68 to 1.24)	0.97 (0.71 to 1.32)	0.853
			4–6/week	0.16 (0.093 to 0.29)	0.43 (0.24 to 0.77)	0.49 (0.28 to 0.87)	0.015
			≥1/day	0.29 (0.15 to 0.54)	0.81 (0.43 to 1.51)	0.92 (0.48 to 1.78)	0.806
	Stroke	Egg consumption					
			<1/week	reference	reference	reference	reference
			1/week	0.46 (0.27 to 0.81)	0.95 (0.54 to 1.67)	1.08 (0.58 ± 1.99)	0.813
			2–3/week	0.25 (0.14 to 0.44)	0.66 (0.36 to 1.23)	0.72 (0.37 to 1.37)	0.313
			4–6/week	0.12 (0.042 to 0.32)	0.40 (0.14 to 1.17)	0.45 (0.15 to 1.39)	0.166
			≥1/day	0.11 (0.025 to 0.45)	0.38 (0.087 to 1.69)	0.48 (0.10 to 2.31)	0.360
	Myocardial infarction	Egg consumption					
			<1/week	reference	reference	reference	reference
			1/week	0.69 (0.26 to 1.85)	1.28 (0.46 to 3.57)	1.30 (0.46 to 3.72)	0.619
			2–3/week	0.28 (0.11 to 0.69)	0.63 (0.22 to 1.77)	0.67 (0.25 to 1.81)	0.428
			4–6/week	0.085 (0.019 to 0.39)	0.24 (0.049 to 1.21)	0.31 (0.059 to 1.58)	0.157
			≥1/day	NA	NA	NA	
	Angina pectoris	Egg consumption					
			<1/week	reference	reference	reference	reference
			1/week	0.90 (0.50 to 1.60)	1.74 (0.96 to 3.13)	1.76 (1.01 to 3.07)	0.048
			2–3/week	0.31 (0.18 to 0.51)	0.74 (0.43 to 1.27)	0.77 (0.44 to 1.35)	0.361
			4–6/week	0.34 (0.15 to 0.77)	1.06 (0.45 to 2.48)	1.27 (0.55 to 2.92)	0.574
			≥1/day	0.39 (0.16 to 0.92)	1.25 (0.51 to 3.05)	1.50 (0.61 to 3.73)	0.379

TC, total cholesterol; TG, triglyceride; HDL-C, high-density lipoprotein cholesterol; LDL-C, low-density lipoprotein cholesterol; NA, not applicable. Data are presented as beta coefficient ± standard error or odds ratio (95% confidence interval). ^a^ Multivariable linear (TC, TG, HDL-C, and LDL-C) or logistic (dyslipidemia, hypertension, diabetes mellitus, stroke, myocardial infarction, and angina pectoris) regression model adjusted for age, body mass index status, total energy intake, protein intake, water intake per body weight, smoking, alcohol drinking, physical activity, education, income, and survey year. ^b^ Log-transformed.

**Table 5:** Association between body composition, waist circumference, and egg consumption in men (food frequency questionnaire).

Sex	Outcome	Characteristics	Crude	Age-Adjusted	Multivariable ^a^	*p*-Value ^a^
Men	Fat mass	Egg consumption				
		<1/week	reference	reference	reference	reference
		1/week	0.60 ± 0.28	0.49 ± 0.29	0.24 ± 0.15	0.101
		2–3/week	0.71 ± 0.26	0.55 ± 0.27	0.30 ± 0.14	0.033
		4–6/week	1.34 ± 0.31	1.13 ± 0.33	0.27 ± 0.18	0.129
		≥1/day	0.99 ± 0.44	0.77 ± 0.46	0.40 ± 0.22	0.078
	Percentage body fat	Egg consumption				
		<1/week	reference	reference	reference	reference
		1/week	0.32 ± 0.26	0.51 ± 0.27	0.33 ± 0.20	0.094
		2–3/week	0.29 ± 0.24	0.56 ± 0.24	0.41 ± 0.18	0.024
		4–6/week	0.64 ± 0.29	0.99 ± 0.30	0.37 ± 0.23	0.104
		≥1/day	0.35 ± 0.43	0.72 ± 0.43	0.49 ± 0.30	0.102
	Fat-free mass	Egg consumption				
		<1/week	reference	reference	reference	reference
		1/week	1.01 ± 0.36	0.22 ± 0.35	−0.19 ± 0.22	0.390
		2–3/week	1.35 ± 0.30	0.21 ± 0.30	−0.25 ± 0.19	0.179
		4–6/week	2.68 ± 0.40	1.17 ± 0.40	0.11 ± 0.26	0.681
		≥1/day	1.94 ± 0.46	0.39 ± 0.47	−0.30 ± 0.33	0.362
	Fat-to-muscle ratio	Egg consumption				
		<1/week	reference	reference	reference	reference
		1/week	0.005 ± 0.004	0.008 ± 0.004	0.005 ± 0.003	0.107
		2–3/week	0.005 ± 0.004	0.009 ± 0.004	0.007 ± 0.003	0.021
		4–6/week	0.01 ± 0.005	0.016 ± 0.005	0.005 ± 0.004	0.163
		≥1/day	0.007 ± 0.007	0.013 ± 0.007	0.009 ± 0.005	0.080
	Waist circumference	Egg consumption				
		<1/week	reference	reference	reference	reference
		1/week	−0.34 ± 0.46	0.32 ± 0.47	−0.066 ± 0.20	0.742
		2–3/week	−0.66 ± 0.42	0.30 ± 0.42	−0.14 ± 0.19	0.470
		4–6/week	0.28 ± 0.53	1.54 ± 0.55	0.088 ± 0.26	0.732
		≥1/day	−0.62 ± 0.67	0.69 ± 0.68	0.082 ± 0.28	0.768

Data are presented as beta coefficient ± standard error. ^a^ Multivariable linear regression model adjusted for age, body mass index status, total energy intake, protein intake, water intake per body weight, smoking, alcohol drinking, physical activity, education, income, and survey year.

**Table 6:** Association between body composition, waist circumference, and egg consumption in women (food frequency questionnaire).

Sex	Outcome	Characteristics	Crude	Age-Adjusted	Multivariable ^a^	*p*-Value ^a^
Women	Fat mass	Egg consumption				
		<1/week	reference	reference	reference	reference
		1/week	−0.33 ± 0.24	0.10 ± 0.24	0.19 ± 0.11	0.087
		2–3/week	−0.40 ± 0.22	0.04 ± 0.23	0.19 ± 0.10	0.068
		4–6/week	−0.16 ± 0.30	0.37 ± 0.31	0.33 ± 0.13	0.009
		≥1/day	−0.82 ± 0.31	−0.28 ± 0.32	0.22 ± 0.14	0.115
	Percentage body fat	Egg consumption				
		<1/week	reference	reference	reference	reference
		1/week	−0.44 ± 0.24	0.19 ± 0.25	0.35 ± 0.18	0.053
		2–3/week	−0.77 ± 0.22	0.04 ± 0.23	0.18 ± 0.16	0.264
		4–6/week	−0.73 ± 0.29	0.27 ± 0.29	0.29 ± 0.20	0.144
		≥1/day	−1.04 ± 0.30	−0.019 ± 0.30	0.39 ± 0.22	0.075
	Fat-free mass	Egg consumption				
		<1/week	reference	reference	reference	reference
		1/week	0.07 ± 0.19	−0.17 ± 0.19	−0.12 ± 0.15	0.421
		2–3/week	0.35 ± 0.17	0.032 ± 0.18	0.045 ± 0.13	0.723
		4–6/week	0.56 ± 0.21	0.18 ± 0.21	0.036 ± 0.16	0.825
		≥1/day	−0.08 ± 0.24	−0.47 ± 0.26	−0.28 ± 0.18	0.126
	Fat-to-muscle ratio	Egg consumption				
		<1/week	reference	reference	reference	reference
		1/week	−0.01 ± 0.006	0.003 ± 0.006	0.007 ± 0.004	0.102
		2–3/week	−0.017 ± 0.005	0.0007 ± 0.005	0.004 ± 0.003	0.269
		4–6/week	−0.014 ± 0.007	0.008 ± 0.007	0.008 ± 0.004	0.068
		≥1/day	−0.023 ± 0.007	−0.0009 ± 0.007	0.009 ± 0.005	0.082
	Waist circumference	Egg consumption				
		<1/week	reference	reference	reference	reference
		1/week	−2.71 ± 0.43	−0.34 ± 0.40	0.17 ± 0.20	0.396
		2–3/week	−3.28 ± 0.38	−0.24 ± 0.39	0.30 ± 0.19	0.119
		4–6/week	−3.80 ± 0.49	−0.06 ± 0.47	0.23 ± 0.24	0.339
		≥1/day	−5.13 ± 0.55	−1.33 ± 0.53	−0.13 ± 0.27	0.618

Data are presented as beta coefficient ± standard error. ^a^ Multivariable linear regression model adjusted for age, body mass index status, total energy intake, protein intake, water intake per body weight, smoking, alcohol drinking, physical activity, education, income, and survey year.

## Data Availability

The data presented in this study are publicly available in the Korea National Health and Nutrition Examination Survey database at https://knhanes.kdca.go.kr/knhanes/sub03/sub03_02_05.do (accessed on 1 November 2021) and https://knhanes.kdca.go.kr/knhanes/eng/index.do (accessed on 1 November 2021).

## Supplementary Materials

The following are available online at https://www.mdpi.com/article/10.3390/jcm10245918/s1, Table S1. General characteristics according to egg consumption in men (24-h dietary recall). Table S2. General characteristics according to egg consumption in women (24-h dietary recall). Table S3. Association between serum cholesterol level, prevalence of dyslipidemia, and egg consumption in men (24-h dietary recall). Table S4. Association between serum cholesterol level, prevalence of dyslipidemia, and egg consumption in women (24-h dietary recall). Table S5. Association between body composition, waist circumference, and egg consumption in men (24-h dietary recall). Table S6. Association between body composition, waist circumference, and egg consumption in women (24-h dietary recall).
