# Supplementary material for: Relationship between Egg Consumption and Body Composition as Well as Serum Cholesterol Level: Korea National Health and Nutrition Examination Survey 2008–2011
Source: J Clin Med. 2021 Dec 16;10(24):5918. doi: 10.3390/jcm10245918 (PMC8706863; doi:10.3390/jcm10245918)
Supplement: Supplementary file 1 [file jcm-10-05918-s001.zip › jcm-1471593-supplementary.pdf]

Table S1. General characteristics according to egg consumption in men (24-hour dietary recall)

Characteristics	Total	<1/day	≥1/day	*p*-Value [a]
Age, years	44.15±0.33	48.45±0.40	40.28±0.35	<0.001
FM, kg	15.52±0.12	15.13±0.15	15.88±0.15	<0.001
PBF, %	21.92±0.13	21.79±0.17	22.04±0.15	0.155
PBF ≥25%	255 (4.62)	111 (3.81)	144 (5.52)	0.002
FFM, kg	51.21±0.13	50.28±0.16	52.05±0.16	<0.001
FtoM	0.30±0.002	0.30±0.002	0.30±0.002	0.222
BMI, kg/m^2	24.03±0.058	23.85±0.077	24.19±0.080	0.001
BMI ≥25 kg/m^2	1,948 (35.28)	968 (33.21)	980 (37.59)	0.001
WC, cm	83.98±0.17	84.07±0.22	83.91±0.23	0.595
WC ≥90 cm	1,454 (26.33)	772 (26.48)	682 (26.16)	0.785
TC, mg/dL	186.67±0.61	186.69±0.85	186.66±0.85	0.985
TC ≥200 mg/dL	1,841 (33.89)	942 (32.96)	899 (34.91)	0.129
TG, mg/dL [b]	124.58±1.01	127.67±1.01	121.87±1.02	0.015
TG ≥150 mg/dL	2,003 (36.81)	1,087 (37.97)	916 (35.53)	0.063
HDL-C, mg/dL	46.18±0.20	45.97±0.29	46.37±0.26	0.287
HDL-C <40 mg/dL	1,831 (33.70)	1,002 (35.06)	829 (32.19)	0.026
LDL-C, mg/dL	112.86±0.96	111.72±1.50	113.74±1.34	0.332
LDL-C ≥130 mg/dL	436 (27.53)	200 (26.25)	236 (28.71)	0.273
Dyslipidemia	454 (8.22)	254 (8.71)	200 (7.67)	0.159
Hypertension	1,240 (22.46)	778 (26,69)	462 (17.72)	<0.001
Diabetes mellitus	473 (8.57)	323 (11.08)	150 (5.75)	<0.001
Stroke	129 (2.34)	87 (2.98)	42 (1.61)	0.001
Myocardial infarction	57 (1.03)	42 (1.44)	15 (0.58)	0.001
Angina pectoris	103 (1.87)	73 (2.50)	30 (1.15)	<0.001
Predicted 10-year risk of a first hard ASCVD event	0.065±0.001	0.088±0.002	0.044±0.001	<0.001
Nutritional intake				
Total energy intake, kcal/day	2327.45±14.96	2126.18±19.01	2509.11±19.79	<0.001
Protein intake, g/day	84.71±0.77	75.14±1.01	93.34±0.99	<0.001
Water intake/body weight, g/kg/day	16.38±0.17	14.78±0.22	17.83±0.25	<0.001
Smoking				<0.001
None	1,244 (22.53)	628 (21.54)	616 (23.63)	
Past	2,029 (36.74)	1,149 (39.42)	880 (33.76)	
Current	2,249 (40.73)	1,138 (39.04)	1,111 (42.62)	
Alcohol drinking				<0.001
<1 time/month	1,420 (25.72)	833 (28.58)	587 (22.52)	
≥1 time/month	4,102 (74.28)	2,082 (71.42)	2,020 (77.48)	
Physical activity				0.376
Low	1,531 (27.73)	785 (26.93)	746 (28.62)	
Moderate	2,153 (38.99)	1,150 (39.45)	1,003 (38.47)	
High	1,838 (33.29)	980 (33.62)	858 (32.91)	

Education				<0.001
0-6 years	896 (16.23)	696 (23.88)	200 (7.67)	
7-12 years	2,317 (41.96)	1,313 (45.04)	1,004 (38.51)	
≥13 years	2,309 (41.81)	906 (31.08)	1,403 (53.82)	
Income				<0.001
Q1	1,305 (23.63)	748 (25.66)	557 (21.37)	
Q2	1,438 (26.04)	785 (26.93)	653 (25.05)	
Q3	1,386 (25.10)	712 (24.43)	674 (25.85)	
Q4	1,393 (25.23)	670 (22.98)	723 (27.73)	
Survey year				<0.001
2008	950 (17.20)	547 (18.77)	403 (15.46)	
2009	2,168 (39.26)	1,179 (40.45)	989 (37.94)	
2010	1,715 (31.06)	830 (28.47)	885 (33.95)	
2011	689 (12.48)	359 (12.32)	330 (12.66)	

FM, fat mass; PBF; percentage body fat; FFM, fat-free mass; FtoM, fat-to-muscle ratio; BMI, body mass index; WC, waist circumference; TC, total cholesterol; TG, triglyceride; HDL-C, high-density lipoprotein cholesterol; LDL-C, low-density lipoprotein cholesterol; ASCVD, atherosclerotic cardiovascular disease. Data are presented as means ± standard error for continuous variables and numbers (%) for categorical variables. [a] *p*-value from linear regression analysis for continuous variables or χ^2 test for categorical variables, comparing differences between two groups. [b] Geometric mean ± standard error.

Table S2. General characteristics according to egg consumption in women (24-hour dietary recall)

**Characteristics**	**Total**	**<1/day**	**≥1/day**	***p*-Value** [a]
Age, years	44.69±0.28	48.75±0.38	40.48±0.29	<0.001
FM, kg	19.01±0.10	19.10±0.13	18.92±0.13	0.267
PBF, %	32.85±0.12	33.07±0.14	32.62±0.14	0.006
PBF ≥30%	261 (3.33)	141 (3.35)	120 (3.30)	0.895
FFM, kg	35.99±0.081	35.86±0.09	36.13±0.11	0.032
FtoM	0.53±0.002	0.53±0.003	0.52±0.003	0.016
BMI, kg/m^2	23.20±0.058	23.47±0.075	22.92±0.080	<0.001
BMI ≥25 kg/m^2	2,226 (28.38)	1,312 (31.19)	914 (25.12)	<0.001
WC, cm	77.68±0.18	78.83±0.23	76.49±0.23	<0.001
WC ≥85 cm	1,978 (25.22)	1,223 (29.08)	755 (20.75)	<0.001
TC, mg/dL	185.80±0.53	188.71±0.69	182.84±0.68	<0.001
TC ≥200 mg/dL	2,675 (35.07)	1,563 (38.46)	1,112 (31.21)	<0.001
TG, mg/dL [b]	91.31±1.01	97.37±1.01	85.53±1.01	<0.001
TG ≥150 mg/dL	1,582 (20.71)	996 (24.47)	586 (16.41)	<0.001
HDL-C, mg/dL	51.30±0.18	50.61±0.23	51.99±0.23	<0.001
HDL-C <50 mg/dL	3,944 (51.71)	2,244 (55.22)	1,700 (47.71)	<0.001
LDL-C, mg/dL	109.96±0.90	111.98±1.30	108.14±1.21	0.030
LDL-C ≥130 mg/dL	464 (26.51)	249 (28.92)	215 (24.18)	0.025
Dyslipidemia	756 (9.64)	468 (11.13)	288 (7.92)	<0.001
Hypertension	1,517 (19.34)	1,037 (24.66)	480 (13.19)	<0.001
Diabetes mellitus	504 (6.43)	348 (8.27)	156 (4.29)	<0.001
Stroke	117 (1.49)	87 (2.07)	30 (0.82)	<0.001
Myocardial infarction	35 (0.45)	26 (0.62)	9 (0.25)	0.014

Angina pectoris	103 (1.31)	72 (1.71)	31 (0.85)	0.001
Predicted 10-year risk of a first hard ASCVD event	0.035±0.001	0.049±0.001	0.021±0.001	<0.001
Nutritional intake				
Total energy intake, kcal/day	1670.27±9.70	1561.29±11.26	1782.84±14.08	<0.001
Protein intake, g/day	59.55±0.46	53.38±0.55	65.92±0.63	<0.001
Water intake/body weight, g/kg/day	15.06±0.16	13.77±0.20	16.39±0.21	<0.001
Smoking				0.065
None	7,165 (91.34)	3,853 (91.61)	3,312 (91.04)	
Past	298 (3.80)	141 (3.35)	157 (4.32)	
Current	381 (4.86)	212 (5.04)	169 (4.65)	
Alcohol drinking				<0.001
<1 time/month	4,742 (60.45)	2,718 (64.62)	2,024 (55.63)	
≥1 time/month	3,102 (39.55)	1,488 (35.38)	1,614 (44.37)	
Physical activity				<0.001
Low	2,588 (32.99)	1,297 (30.84)	1,291 (35.49)	
Moderate	3,363 (42.87)	1,844 (43.84)	1,519 (41.75)	
High	1,893 (24.13)	1,065 (25.32)	828 (22.76)	
Education				<0.001
0-6 years	2,275 (29.00)	1,729 (41.11)	546 (15.01)	
7-12 years	3,140 (40.03)	1,582 (37.61)	1,558 (42.83)	
≥13 years	2,429 (30.97)	895 (21.28)	1,534 (42.17)	
Income				<0.001
Q1	1,898 (24.20)	1,085 (25.80)	813 (22.35)	
Q2	2,021 (25.76)	1,118 (26.58)	903 (24.82)	
Q3	2,006 (25.57)	1,067 (25.37)	939 (25.81)	
Q4	1,919 (24.46)	936 (22.25)	983 (27.02)	
Survey year				<0.001
2008	1,489 (18.98)	878 (20.87)	611 (16.79)	
2009	2,975 (37.93)	1,614 (38.37)	1,361 (37.41)	
2010	2,396 (30.55)	1,225 (29.13)	1,171 (32.19)	
2011	984 (12.54)	489 (11.63)	495 (13.61)	

FM, fat mass; PBF; percentage body fat; FFM, fat-free mass; FtoM, fat-to-muscle ratio; BMI, body mass index; WC, waist circumference; TC, total cholesterol; TG, triglyceride; HDL-C, high-density lipoprotein cholesterol; LDL-C, low-density lipoprotein cholesterol; ASCVD, atherosclerotic cardiovascular disease. Data are presented as means ± standard error for continuous variables and numbers (%) for categorical variables. [a] *p*-value from linear regression analysis for continuous variables or χ^2 test for categorical variables, comparing differences between two groups. [b] Geometric mean ± standard error.

Table S3. Association between serum cholesterol level, prevalence of dyslipidemia, and egg consumption in men (24-hour dietary recall)

Sex	Outcome	Characteristics		Crude	Age-adjusted	Multivariable [a]	*p*-Value [a]
Men	TC	Egg consumption					
			<1/day	reference	reference	reference	reference
			≥1/day	−0.02±1.19	2.16±1.22	0.60±1.22	0.622

TG [b]	Egg consumption					
		<1/day	reference	reference	reference	reference
		≥1/day	−0.046±0.019	0.001±0.019	−0.025±0.019	0.185
HDL-C	Egg consumption					
		<1/day	reference	reference	reference	reference
		≥1/day	0.40±0.37	−0.09±0.38	0.039±0.37	0.915
LDL-C	Egg consumption					
		<1/day	reference	reference	reference	reference
		≥1/day	2.01±2.07	4.01±2.11	3.54±2.08	0.089
Dyslipidemia	Egg consumption					
		<1/day	reference	reference	reference	reference
		≥1/day	0.79 (0.63 to 0.99)	1.13 (0.88 to 1.45)	0.98 (0.76 to 1.26)	0.855
Hypertension	Egg consumption					
		<1/day	reference	reference	reference	reference
		≥1/day	0.60 (0.51 to 0.71)	1.14 (0.94 to 1.38)	1.05 (0.86 to 1.28)	0.639
Diabetes mellitus	Egg consumption					
		<1/day	reference	reference	reference	reference
		≥1/day	0.46 (0.36 to 0.59)	0.80 (0.60 to 1.05)	0.81 (0.60 to 1.09)	0.169
Stroke	Egg consumption					
		<1/day	reference	reference	reference	reference
		≥1/day	0.50 (0.31 to 0.81)	1.05 (0.62 to 1.77)	1.26 (0.72 to 2.23)	0.421
Myocardial infarction	Egg consumption					
		<1/day	reference	reference	reference	reference
		≥1/day	0.49 (0.22 to 1.07)	0.94 (0.44 to 1.98)	0.98 (0.43 to 2.23)	0.965
Angina pectoris	Egg consumption					
		<1/day	reference	reference	reference	reference
		≥1/day	0.40 (0.23 to 0.69)	0.80 (0.44 to 1.47)	0.71 (0.40 to 1.27)	0.248

TC, total cholesterol; TG, triglyceride; HDL-C, high-density lipoprotein cholesterol; LDL-C, low-density lipoprotein cholesterol. Data are presented as beta coefficient ± standard error or odds ratio (95% confidence interval). [a] Multivariable linear (TC, TG, HDL-C, and LDL-C) or logistic (dyslipidemia, hypertension, diabetes mellitus, stroke, myocardial infarction, and angina pectoris) regression model adjusted for age, body mass index status, total energy intake, protein intake, water intake per body weight, smoking, alcohol drinking, physical activity, education, income, and survey year. [b] Log-transformed.

Table S4. Association between serum cholesterol level, prevalence of dyslipidemia, and egg consumption in women (24-hour dietary recall)

Sex	Outcome	Characteristics	Crude	Age-adjusted	Multivariable [a]	*p*-Value [a]
Women	TC	Egg consumption				
		<1/day	reference	reference	reference	reference
		≥1/day	−5.88±0.88	0.53±0.88	0.54±0.87	0.530
	TG [b]	Egg consumption				
		<1/day	reference	reference	reference	reference
		≥1/day	−0.13±0.016	−0.022±0.015	−0.005±0.014	0.679
	HDL-C	Egg consumption				
		<1/day	reference	reference	reference	reference
		≥1/day	1.38±0.30	0.14±0.31	−0.18±0.31	0.558
	LDL-C	Egg consumption				
		<1/day	reference	reference	reference	reference
		≥1/day	−3.83±1.76	0.72±1.73	1.74±1.66	0.295
	Dyslipidemia	Egg consumption				
		<1/day	reference	reference	reference	reference
		≥1/day	0.63 (0.53 to 0.76)	1.10 (0.89 to 1.34)	1.03 (0.84 to 1.27)	0.765
	Hypertension	Egg consumption				
		<1/day	reference	reference	reference	reference
		≥1/day	0.49 (0.42 to 0.56)	1.16 (0.98 to 1.38)	1.20 (1.00 to 1.43)	0.044
	Diabetes mellitus	Egg consumption				
		<1/day	reference	reference	reference	reference
		≥1/day	0.58 (0.46 to 0.74)	1.11 (0.85 to 1.44)	1.14 (0.88 to 1.48)	0.304
	Stroke	Egg consumption				
		<1/day	reference	reference	reference	reference
		≥1/day	0.33 (0.20 to 0.53)	0.71 (0.42 to 1.19)	0.73 (0.42 to 1.27)	0.264
	Myocardial infarction	Egg consumption				
		<1/day	reference	reference	reference	reference
		≥1/day	0.39 (0.15 to 0.99)	0,76 (0.28 to 2.07)	0.89 (0.34 to 2.37)	0.818
	Angina pectoris	Egg consumption				
		<1/day	reference	reference	reference	reference
		≥1/day	0.51 (0.30 to 0.85)	1.00 (0.57 to 1.77)	1.10 (0.65 to 1.85)	0.725

TC, total cholesterol; TG, triglyceride; HDL-C, high-density lipoprotein cholesterol; LDL-C, low-density lipoprotein cholesterol; NA, not applicable. Data are presented as beta coefficient ± standard error or odds ratio (95% confidence interval). [a] Multivariable linear (TC, TG, HDL-C, and LDL-C) or logistic (dyslipidemia, hypertension, diabetes mellitus, stroke, myocardial infarction, and angina pectoris) regression model adjusted for age, body mass index status, total energy intake, protein intake, water intake per body weight, smoking, alcohol drinking, physical activity, education, income, and survey year. [b] Log-transformed.

Table S5. Association between body composition, waist circumference, and egg consumption in men (24-hour dietary recall)

Sex	Outcome	Characteristics	Crude	Age-adjusted	Multivariable [a]	*p*-Value [a]
Men	Fat mass	Egg consumption				
		<1/day	reference	reference	reference	reference
		≥1/day	0.75±0.19	0.58±0.20	0.093±0.10	0.361
	Percentage body fat	Egg consumption				
		<1/day	reference	reference	reference	reference
		≥1/day	0.25±0.18	0.50±0.19	0.17±0.13	0.192
	Fat-free mass	Egg consumption				
		<1/day	reference	reference	reference	reference
		≥1/day	1.77±0.20	0.69±0.21	−0.074±0.15	0.618
	Fat-to-muscle ratio	Egg consumption				
		<1/day	reference	reference	reference	reference
		≥1/day	0.003±0.003	0.007±0.003	0.002±0.002	0.391
	Waist circumference	Egg consumption				
		<1/day	reference	reference	reference	reference
		≥1/day	−0.15±0.29	0.72±0.30	−0.12±0.14	0.396

Data are presented as beta coefficient ± standard error. [a] Multivariable linear regression model adjusted for age, body mass index status, total energy intake, protein intake, water intake per body weight, smoking, alcohol drinking, physical activity, education, income, and survey year.

Table S6. Association between body composition, waist circumference, and egg consumption in women (24-hour dietary recall)

Sex	Outcome	Characteristics	Crude	Age-adjusted	Multivariable [a]	*p*-Value [a]
Women	Fat mass	Egg consumption				
		<1/day	reference	reference	reference	reference
		≥1/day	−0.19±0.17	0.097±0.17	0.087±0.072	0.230
	Percentage body fat	Egg consumption				
		<1/day	reference	reference	reference	reference
		≥1/day	−0.45±0.16	0.10±0.16	0.11±0.11	0.330
	Fat-free mass	Egg consumption				
		<1/day	reference	reference	reference	reference
		≥1/day	0.27±0.12	0.025±0.12	−0.024±0.096	0.801
	Fat-to-muscle ratio	Egg consumption				
		<1/day	reference	reference	reference	reference
		≥1/day	−0.009±0.003	0.002±0.003	0.002±0.002	0.310
	Waist circumference	Egg consumption				
		<1/day	reference	reference	reference	reference
		≥1/day	−2.33±0.29	−0.19±0.27	−0.025±0.13	0.839

Data are presented as beta coefficient ± standard error. [a] Multivariable linear regression model adjusted for age, body mass index status, total energy intake, protein intake, water intake per body weight, smoking, alcohol drinking, physical activity, education, income, and survey year.
